# Do Epigeal Termite Mounds Increase the Diversity of Plant Habitats in a Tropical Rain Forest in Peninsular Malaysia?

**DOI:** 10.1371/journal.pone.0019777

**Published:** 2011-05-19

**Authors:** Lydia Beaudrot, Yanjun Du, Abdul Rahman Kassim, Marcel Rejmánek, Rhett D. Harrison

**Affiliations:** 1 Graduate Group in Ecology, University of California Davis, Davis, California, United States of America; 2 International Field Biology Course 2008, Arnold Arboretum, Center for Tropical Forest Science, Pasoh Forest Reserve, Malaysia; 3 Institute of Botany, Chinese Academy of Sciences, Beijing, China; 4 Forest Research Institute Malaysia, Selangor, Malaysia; 5 Department of Evolution and Ecology, University of California Davis, Davis, California, United States of America; 6 Xishuangbanna Tropical Botanical Garden, Chinese Academy of Sciences, Mengla, Yunnan Province, China; Duke University, United States

## Abstract

The extent to which environmental heterogeneity can account for tree species coexistence in diverse ecosystems, such as tropical rainforests, is hotly debated, although the importance of spatial variability in contributing to species co-existence is well recognized. Termites contribute to the micro-topographical and nutrient spatial heterogeneity of tropical forests. We therefore investigated whether epigeal termite mounds could contribute to the coexistence of plant species within a 50 ha plot at Pasoh Forest Reserve, Malaysia. Overall, stem density was significantly higher on mounds than in their immediate surroundings, but tree species diversity was significantly lower. Canonical correspondence analysis showed that location on or off mounds significantly influenced species distribution when stems were characterized by basal area. Like studies of termite mounds in other ecosystems, our results suggest that epigeal termite mounds provide a specific microhabitat for the enhanced growth and survival of certain species in these species-rich tropical forests. However, the extent to which epigeal termite mounds facilitate species coexistence warrants further investigation.

## Introduction

Understanding the mechanisms of biodiversity maintenance remains a fundamental challenge in ecology and is of particular interest for understanding community assembly processes in species-rich tropical forests [1], [2], [3]. A large number of hypotheses have been put forward, but classic niche differentiation has received more attention than others. According to niche differentiation, environmental variation in space and time increases the available niche space through trade-offs in maximizing fitness to different sets of conditions, thereby increasing the number of species capable of successfully competing for limited resources [4], [5], [6], [7].

The spatial scale at which niche differences are examined can greatly affect our understanding of the role of niche differentiation in driving biodiversity patterns [8], [9]. Much emphasis has been given to the role of edaphic factors in explaining large (>1 km) [8], [10], [11] and medium (20 m–1 km) [1], [2], [3], [6], [7], [12], [13] scale variation in floristic patterns in tropical communities. Several studies have also addressed the role of small-scale environmental variation in affecting seedling establishment and performance [14], [15], [16], [17]. However, few studies have investigated floristic patterns at these micro-habitat (<10 m) scales within tropical forests. Termites are a recognized agent of small-scale ecosystem change that affect the same soil conditions, such as nutrients, moisture and texture that are known to affect floristic patterns at larger scales. Hence, to understand the role of local-scale processes on species coexistence, we investigated the influence of termite mounds on plant communities in a species-rich forest in Peninsular Malaysia.

As ecosystem engineers, termites contribute to micro-topographical and nutrient spatial heterogeneity in tropical forests over time [18], [19], [20]. Termite activities during nest building in particular change the structure, drainage and chemical composition of soils [21], [22], [23]. Epigeal termite mounds are constructed below ground into a raised nest above ground. By mixing soil with decomposed leaf-litter, termites raise nutrient levels, particularly Ca, K and Mg, of the soil used in mound building [24]. Moreover, persistence of abandoned epigeal mounds has been estimated at 20–25 years [25] and therefore mounds provide a nutrient enriched microhabitat for an extended period of time [26].

There is strong evidence that mound-building organisms, such as gophers and ants, affect the dynamics and spatial patterning of plant communities [27], [28], [29]. Moreover, there is growing evidence that termite mounds affect plant communities in the systems in which they have been studied. In North-eastern Australia, plant biomass changes with distance from termite mounds [30]. Termite mounds have been shown to be favourable sites for woody plant recruitment in savannah woodlands in Burkina Faso [31] and have been shown to contribute to plant diversity in savannah habitats in Uganda [32]. Studies of mound building termites in Australia and Amazonia have reported improved plant growth on mounds attributed to higher soil nutrient levels [33], [34], [35]. Reports from strongly seasonal forests in Thailand describe strikingly different growth of several plant species on termite mounds than in nearby areas [36] and savanna termite mounds in Africa have been documented to host specialist plant species [37]. Epigeal termite mounds may also affect plant dynamics and community composition within tropical rain forests, but to date there are no quantitative reports of which we are aware.

In this study, we asked whether epigeal mounds affect tree density, diversity, and species distribution. If epigeal mounds have altered physical and chemical soil properties in comparison with their surroundings, then these mounds might be expected to support specialist plant species environmentally filtered from the overall species pool. We therefore tested the extent to which termite mounds influence tree density, diversity and community composition. We evaluated our results in light of the larger question of whether termite mounds support a distinct sub-community of plants in Pasoh Forest Reserve and in doing so contribute to the overall biodiversity.

## Methods

### Study Site

This study was conducted in Pasoh Forest Reserve, a lowland dipterocarp forest located approximately 140 km southeast of Kuala Lumpur, Peninsular Malaysia (2°58′ N, 102°18′ E, alt. 75–190 m). Mean annual rainfall at Pasoh Forest Reserve is approximately 2000 mm with two wet seasons and mean monthly rainfall >100 mm in all months (for more site information see [38]). In 1987 a permanent 50-hectare plot was established. Trees in the plot with diameter-at-breast-height (dbh) greater than 1 cm were tagged, identified, mapped and dbh was measured. The plot has since been censused at five-year intervals. All data for this study were collected within the 50 ha permanent plot during July and August of 2008.

Soils within the Pasoh plot have been categorized into four general types based on eleven soil series identified from samples taken at 40×40 meter resolution. The soil types are wet alluvium (WA), dry alluvium (DA), shale (SH) and laterite (LA). A detailed description of the soil data collection and identification methods has been published elsewhere [39], [40].

**Figure 1:**
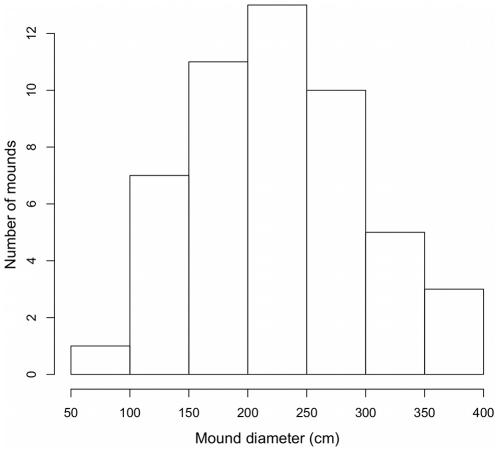
Histogram of mound diameters. The smaller of the recorded North-South and East-West diameters was used for each mound. The large size of the mound diameters suggests they were constructed by *Macrotermes*.

Considerable research on the termite fauna of the Pasoh Forest Reserve has been carried out [41], [42], [43], [44], [45]. Termite species found within Pasoh that commonly build epigeal mounds include *Macrotermes carbonarius*, *Dicuspiditermes nemorosus*, and *Homallotermes foraminifer*. However, *M. carbonarius* build much larger epigeal mounds than other species [43]. Our study focused on the trees growing only on larger epigeal mounds (>80 cm minimum N-S or E-W diameter; Figure 1) that had presumably belonged to *M. carbonarius*. However, direct confirmation of the termite species responsible for the mounds studied was not possible as in most cases the mounds were no longer occupied.

### Data Collection

We surveyed forty 20 m×20 m plots for epigeal termite mounds. We used random start-points but stratified sampling among the four soil types. Ten plots were located in each of the four soil types. In each plot, we recorded the number of termite mounds and their structure (height, north-south diameter and east-west diameter at the base; *cf* [24]).

**Figure 2:**
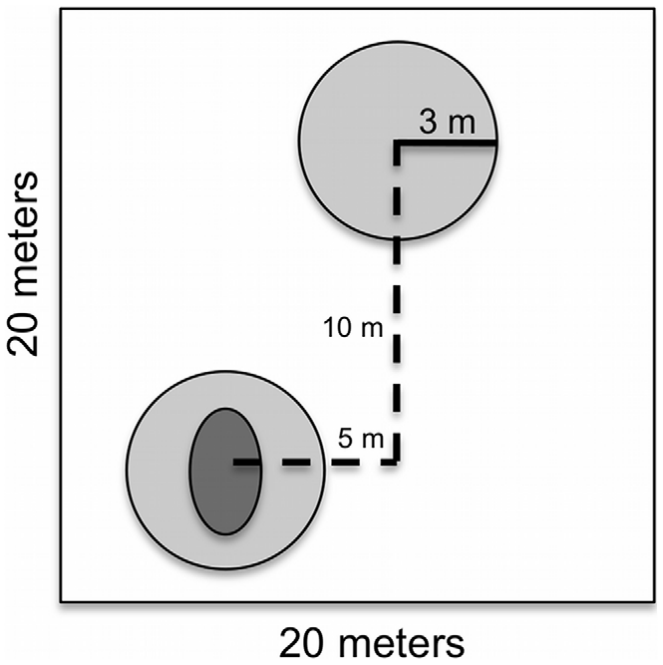
Sampling Design. 20×20 meter plot with a 3-m radius subplot containing a mound and a paired 3-m radius subplot without a mound. Dark gray represents the area sampled containing a mound. Light gray represents the area sampled without a mound. Subplots with mounds were sampled in the field in 2008. Subplots without mounds and all species data were extracted from the 2005 Pasoh census data.

The soils of the areas immediately surrounding mounds are likely to be influenced by erosion of the mound soil and by subterranean portions of the mounds. However, the extent of these effects is unknown and it is unlikely that areas entirely free of termite influence could be found given the high density of nests in Pasoh. Moreover, randomly selected areas between mounds are more likely to differ from mounds in terms of other unmeasured microhabitat factors, such as light conditions, than the area immediately surrounding the mound. Hence, to assess the influence of mounds on tree recruitment we conducted two tests. In the first, we compared mounds with their immediate surroundings. In the second, we compared 3-m radius subplots with mounds to paired randomly selected 3-m radius subplots without mounds located within the same 20×20 m plot. In the field, we recorded the tree identification tags for all trees growing on the mound and off the mound within a 3 m radius of the mound center. Then we extracted the paired 3-m subplots without a mound from the Pasoh database. For the paired plots, we do not have an estimate of how many individuals may have been present in 2005 but not in 2008. A diagram depicting our sampling design is shown in Figure 2.

It is conceivable that some stems had established before the formation of the mound. However, *M. carbonarius* is known to prevent trees establishing on mounds while the mounds are occupied. Our observations also indicated the stems were growing on top of the mounds rather than through them. We therefore believe this source of error to be minimal.

Using the tree tag data, we extracted the corresponding information on tree species identity and dbh from the 2005 census in the unpublished long-term Pasoh dataset, which was the most recent Pasoh census data available. We defined stems as tagged individuals recorded during our 2008 data collection and in the plot census from 2005. Because stems are only tagged during the 5-year censuses, the only individuals for which we did not have the corresponding data were stems that had reached the 1 cm dbh threshold after the 2005 census. We do not have an estimate of how many individuals this may have been.

For a subset of the mounds surveyed (N = 8) we recorded morpho-species abundance of seedlings growing on termite mounds and in a 0.75 m×0.75 m quadrat 3 m north of the termite mound centers. We defined seedlings as stems less than one meter in height. We were unable to collect comparable data on the remaining mounds because of time constraints.

### Data Analysis

We used a generalized linear model to examine the relationship between soil type and termite mound frequency. We modeled the number of mounds per plot as a function of the soil types using a Poisson distribution with the glm function in the bblme package in R2.8.1 [46]. We used a one-way ANOVA to analyze the relationship between soil types and mound structure (height, surface area of the ground covered by the mound estimated as an ellipsoid, and above-ground volume estimated as a half-ellipsoid). We square root transformed the surface area and volume estimates to normalize the distributions and stabilize the variances around the means. We used paired t-tests for comparisons of stem density (stems per m2) and species diversity. For both, the distributions of the differences of the paired values met the assumption of near normality. We estimated species diversity using Simpson's diversity index based on stem density. Simpson's diversity index (SI) can be applied to either the density of individuals per species or the basal area per species. It is calculated using the equation: SI = 1−Σp_i_^2^ where p_i_ is the proportional abundance of species i. Simpson's diversity index is relatively insensitive to sample size and therefore is not likely influenced by the number of stems in a sample. Analyses were conducted in R2.8.1 [46].

We also examined the effect of stem size on variation in density, diversity and composition in mound and non-mound sites. We partitioned the data into juveniles (1–10 cm dbh) and adult trees (≥10 cm dbh) [13] and repeated the analyses described above, using a Bonferroni correction for multiple comparisons (N = 8; p = 0.05/8 = 0.006).

We conducted two sets of direct gradient ordinations using partial canonical correspondence analysis with the software CANOCO 4.5 for Windows [47]. Sites were defined as mounds and areas immediately surrounding mounds within a 3-meter radius of the mound center. In the first set of ordination analyses, species were quantified by density. In the second set, species were quantified by total basal area. Each set of ordination analyses investigated juvenile stems, adult stems and all stems. We used soil type and location on or off mound as the nominal environmental variables. We used surface area of the mound and of the ground as co-variables to account for the variation in area between mounds and the area surrounding mounds. We estimated mound surface area as a half ellipsoid based on diameter and height measurements. We used the raw data with no standardizations or transformations. We used linear combinations of environmental variables (LC) rather than weighted average (WA) site scores. We ran Monte Carlo simulations with 999 unrestricted permutations with a Bonferroni correction for multiple comparisons (p = 0.05/6 = 0.008).

Comparisons of per species growth or mortality for mound and non-mound sites were not possible, despite our substantial sampling effort, because of the low per species sample sizes. These limitations also precluded identifying mound and non-mound specialist species.

## Results

### Influence of soil type on mound characteristics

The number of termite mounds per plot ranged from 0–3 mounds, and we recorded 50 mounds in total for the 1.6 hectares we surveyed (40 20×20 meter plots). This is equivalent to an overall density of 31.25 epigeal mounds per hectare. The number of mounds per plot varied among soil types (mounds per plot: wet alluvium 14; dry alluvium 9; shale 17; laterite 10). However soil types were not significant predictors of the number of mounds per plot (Poisson generalized linear model: DF = 36; α = 9.03e-14, p = 1.00; β1 = 3.37e-1, p = 0.416; β2 = −1.05e-1, p = 0.819; β3 = 5.31e-1, p = 0.183). Mound heights ranged from 27 cm to 153 cm, mound diameters ranged from 80 cm to 600 cm, ground surface area covered by the mound ranged from 0.65 m2 to 14.14 m2 when estimated as an ellipse and mound volume ranged from 0.62 m3 to 18.74 m3 when estimated as a half-ellipsoid. The mean non-mound area sampled was 22.56 m^2^ and the mean mound area sampled was 5.72 m^2^. We did not find significant differences between soil types in mound height (F3,46 = 0.736, *p* = 0.536), estimated ground surface area covered by the mound (F3,46 = 0.287, *p* = 0.834), or estimated mound volume (F3,46 = 0.092, *p* = 0.964). Thus, soil type did not have a significant influence on mound density, size, or shape.

### Seedling and tree density

We recorded 421 seedlings from eight mounds and paired non-mound quadrats. Of these, 389 seedlings were located on the mounds and 32 seedlings were located in the seedling quadrats 3 meters north of the mound center. In addition, we recorded tree identification tags for 579 stems >1 cm dbh in 3 m subplots containing mounds. Of these, 165 stems were located on the mounds (149 juveniles and 16 adults). The remaining 414 stems (372 juveniles and 42 adults) were found in the area off of the mounds but within the 3-meter radius of the mound center. We have included data on the species and 2005 dbh measurements of the 16 adult trees located on the mounds (Table 1). Because we do not know the relative ages of the trees or mounds, we do not know how many trees became established after the mounds had been built. For this reason, we provide separate analyses for all stems and for juvenile and adult stems considered separately.

**Table 1:** Species identification and dbh measurements of the adult trees growing on mounds.

Family	Genus	Species	DBH (cm)
Annonaceae	*Enicosanthum*	*fuscum*	10
Verbenaceae	*Callicarpa*	*maingayi*	10.5
Rubiaceae	*Aidia*	*wallichiana*	10.9
Fagaceae	*Lithocarpus*	*curtisii*	11.5
Tiliaceae	*Schoutenia*	*accrescens*	13.8
Rubiaceae	*Aidia*	*wallichiana*	14.8
Annonaceae	*Anaxagorea*	*javanica*	15.3
Myristicaceae	*Knema*	*intermedia*	15.4
Myrtaceae	*Eugenia*	*filiformis*	15.7
Flacourtiaceae	*Homalium*	*longifolium*	15.8
Ulmaceae	*Gironniera*	*parvifolia*	19.1
Rubiaceae	*Aidia*	*wallichiana*	22.7
Sapindaceae	*Nephelium*	*costatum*	25.6
Leguminosae	*Cynometra*	*malaccensis*	35
Leguminosae	*Cynometra*	*malaccensis*	38.1
Apocynaceae	Alstonia	angustiloba	81.8

**Figure 3:**
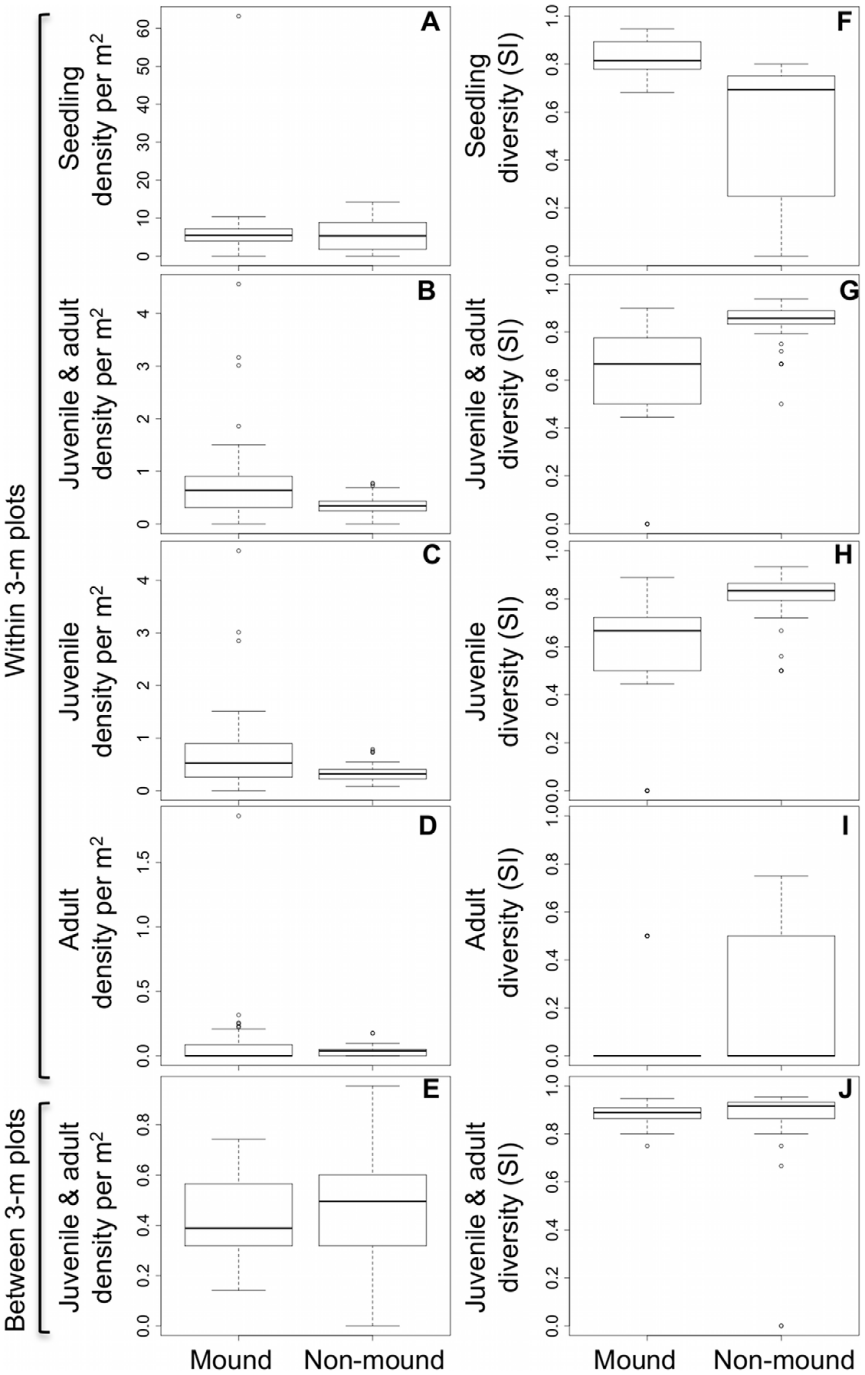
Density and diversity of stems in mound and non-mound areas. (**a**) Seedling density was not significantly different between mounds and off-mound seedling plots (paired t-test; t = 0.874, df = 7, p = 0.411) (**b**) For all trees (>1 cm dbh) stem density was significantly higher on mounds than around mounds (sample estimate mean of differences = 0.427 m−2, t = 3.54, df = 48, p<0.001). (**c**) There were significantly more juvenile stems (1–10 cm dbh) on mounds than around mounds (sample estimate mean of differences = 0.367 m−2, t = 3.07, df = 48, p = 0.004). (**d**) Adult stem density (>10 cm dbh) was not significantly different on mounds and around mounds (sample estimate mean of the differences = 0.048, t = 1.20, df = 48, p = 0.235). (**e**) For all trees stem density in 3-m subplots with mounds was not significantly different from paired 3-m subplots without mounds (sample estimate mean of the differences = −0.045, t = −1.11, df = 48, p = 0.273; Figure 3e) (**f**) There was a significantly higher diversity of seedling morpho-species found on mounds than in paired seedling plots (paired t-test; t = 2.54, df = 7, p-value = 0.039) (**g**) The diversity of all stems was significantly lower on mounds compared to around mounds (sample estimate mean of the differences = −0.218, t = −5.39, df = 48, *p*<0.001). (**h**) The diversity of juvenile stems was significantly lower on mounds than around mounds (sample estimate mean of the differences = −0.252, t = −6.49, df = 48, p<0.001). (**i**) Adult stem diversity was not significantly different between mound and nonmound areas (sample estimate mean of the differences = −0.054, t = −1.16, df = 48, p = 0.252). (**j**) The diversity of all stems in 3-m radius plots with mounds with paired 3-m radius plots without mounds did not differ significantly (0.889 vs 0.856, respectively; sample estimate mean of the differences = 0.026, t = 0.943, df = 48, p = 0.351). The lower and upper edges of box represent the 25th and 75th percentile of observations, respectively. The dark line within the box represents the mean. The whiskers depict 1.5 times the inter-quartile range. All data points outside the whiskers are shown as outliers. With a Bonferroni correction for multiple comparisons for analyses of juveniles and adults, p is significant at the level of 0.006 (p = α/n).

Seedling density per square meter was not significantly different between mound and non-mound areas (paired t-test; t = 0.874, df = 7, p = 0.411) (Fig. 3a). However, mean stem density for all trees >1 cm dbh (0.794 stems m−2 for mounds and 0.367 stems m−2 for nonmounds) was significantly higher on mounds than in their immediate surroundings (sample estimate mean of differences = 0.427 m−2, t = 3.54, df = 48, p<0.001; Figure 3b). Mean stem density was also significantly higher on mounds than in their immediate surroundings for juvenile trees considered alone (0.710 stems m−2 for mounds and 0.343 stems m−2 for non-mounds; sample estimate mean of differences = 0.367 m−2, t = 3.07, df = 48, p = 0.004; Figure 3c), but not for adult trees (0.0861 stems m−2 for mounds and 0.034 stems m−2 for nonmounds; sample estimate mean of the differences = 0.048, t = 1.20, df = 48, p = 0.235; Figure 3d). When we compared the density of all stems in 3-m radius mound plots with paired 3-m radius plots without mounds the differences were not significant (0.418 stems m−2 vs 0.463 stems m−2, respectively; sample estimate mean of the differences = −0.045, t = −1.11, df = 48, p = 0.273; Figure 3e).

### Seedling and tree diversity

Seedling diversity on mounds was significantly higher than in the paired non-mound areas (paired t-test; t = 2.54, df = 7, p = 0.039) (Fig. 3f). However, the reverse was true for tree diversity. Mean tree species diversity for all stems was 0.617 for mounds and 0.835 for areas immediately surrounding mounds (sample estimate mean of the differences = −0.218, t = −5.39, df = 48, *p*<0.001; Figure 3g). Comparing size classes separately, the diversity of juvenile trees on mounds (0.552) was significantly lower than for areas immediately surrounding mounds (0.804) (sample estimate mean of the differences = −0.252, t = −6.49, df = 48, p<0.001; Figure 3h), but for adult trees the difference was not significant (0.122 vs 0.177, respectively; sample estimate mean of the differences = −0.054, t = −1.16, df = 48, p = 0.252; Figure 3i). When comparing the diversity of all stems in 3-m radius plots with mounds with paired 3-m radius plots without mounds, we found no significant differences (0.889 vs 0.856, respectively; sample estimate mean of the differences = 0.026, t = 0.943, df = 48, p = 0.351; Figure 3j).

### Effects of termite mounds on species composition

**Figure 4:**
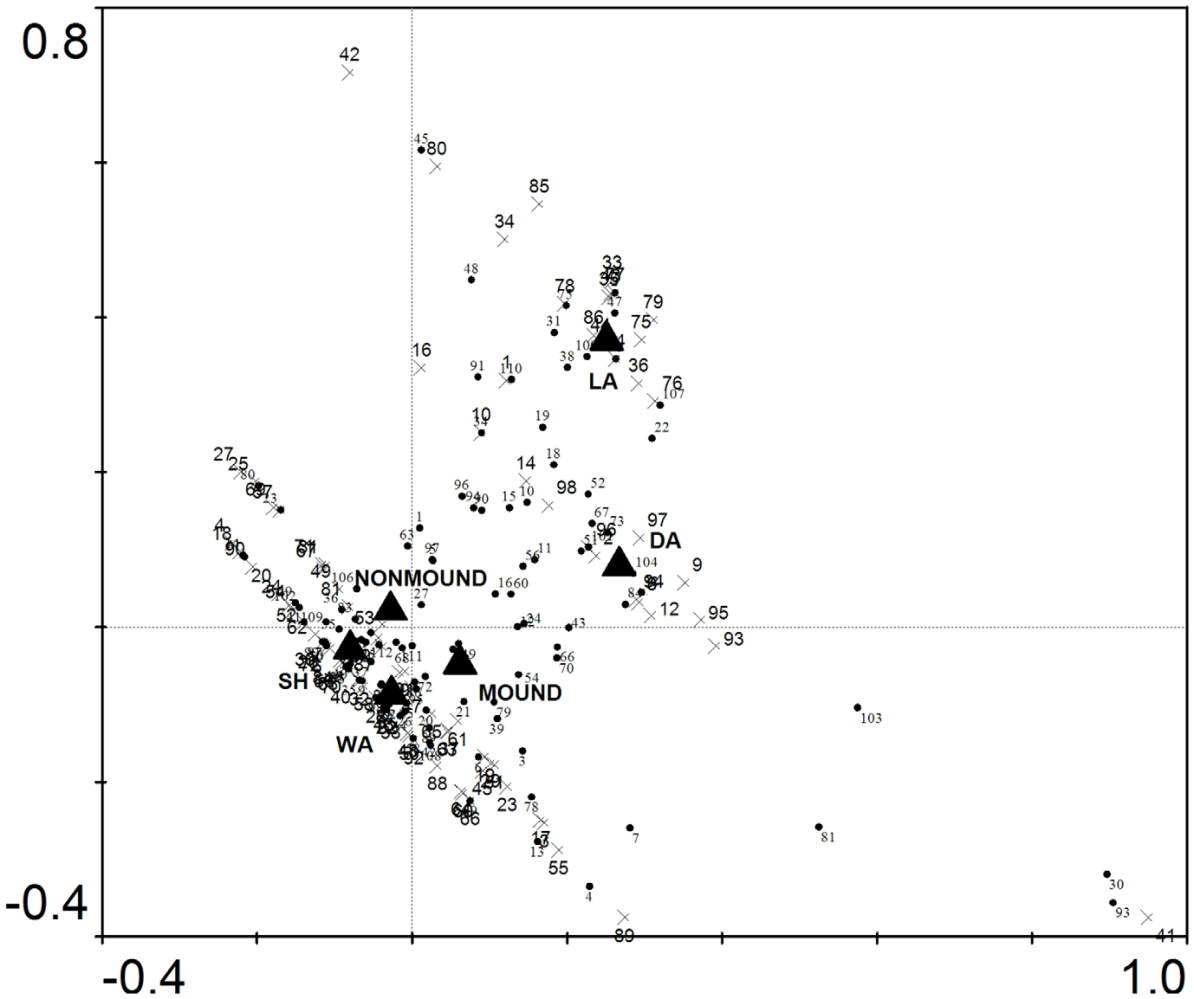
Canonical correspondence analysis triplot of sites, species (dots) and centroids of nominal environmental variables (triangles) based on total basal area of all stems (>1 cm dbh) found on mounds and areas immediately around mounds. The direction and strength of the environmental variables are indicated by the distance from the origin (see Table 2 for significance values). Species are shown with black circles. Mound sites are shown with green triangles and non-mound sites are shown with blue crosses. Species located near the origin are poorly predicted by any variable whereas species far from the origin are best predicted by the nominal variable in close proximity. Soil types are wet alluvium (WA), dry alluvium (DA), shale (SH) and latterite (LA).

**Figure 5:**
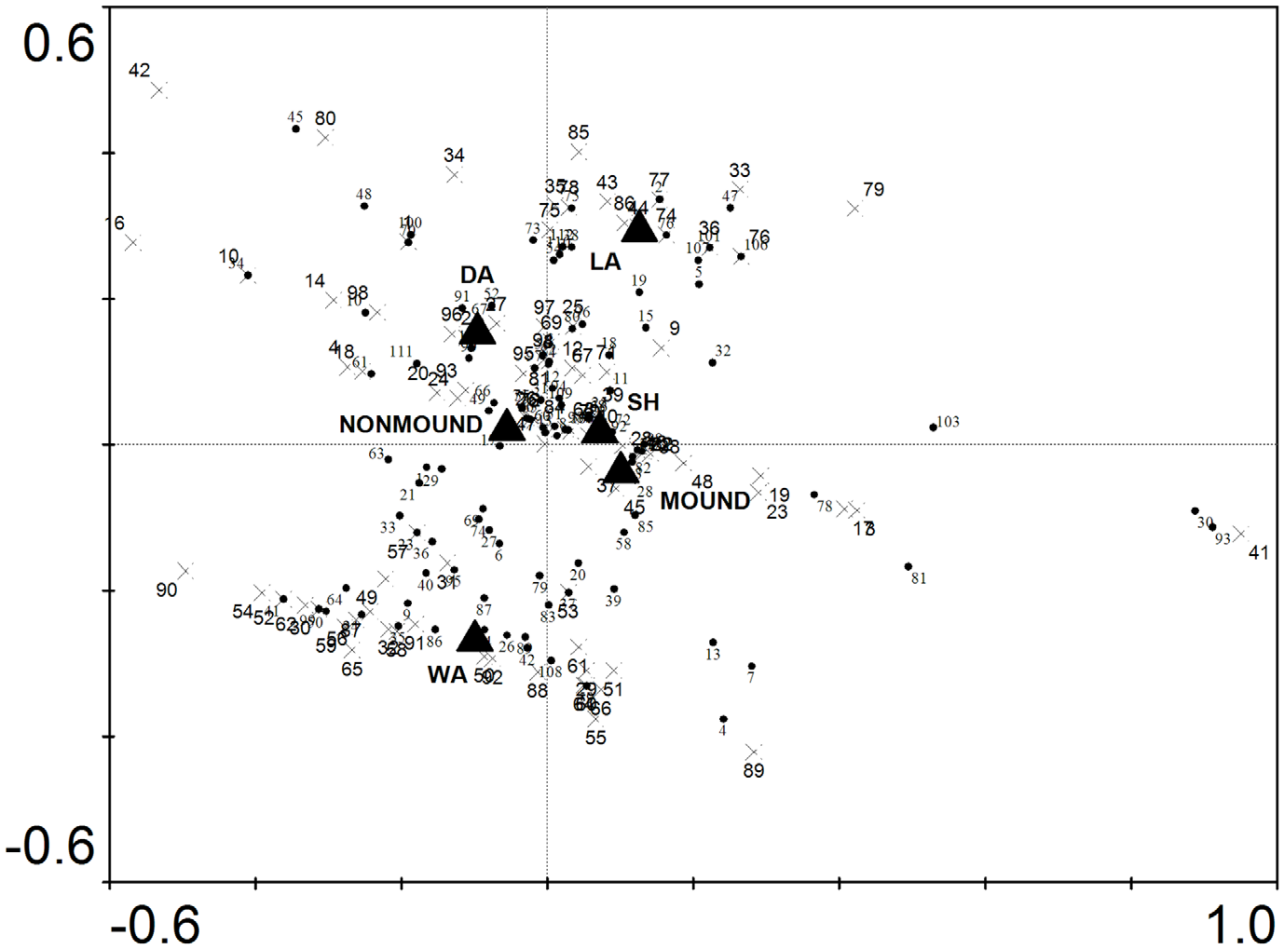
Canonical correspondence analysis triplot of sites, species (dots) and centroids of nominal environmental variables (triangles) based on total basal area of juvenile stems (1–10 cm dbh) found on mounds and areas immediately around mounds. The direction and strength of the environmental variables are indicated by the distance from the origin (see Table 2 for significance values). Species are shown with black circles. Mound sites are shown with green triangles and non-mound sites are shown with blue crosses. Species located near the origin are poorly predicted by any variable whereas species far from the origin are best predicted by the nominal variable in close proximity. Soil types are wet alluvium (WA), dry alluvium (DA), shale (SH) and latterite (LA).

In the partial canonical correspondence analysis of all stems (Table 2a), juvenile stems (Table 2b) and adult stems (Table 2c) we found that when species were quantified by tree density and surface area was used as a covariate, community composition did not show any significant responses to location on or off of mounds. However, when species were quantified by basal area and surface area was used as a covariate, location on or off mounds was a highly significant predictor of variation in community composition for all stems (Table 2d; Figure 4) and for juvenile stems alone (Table 2e; Figure 5), but not for adult stems (Table 2f). In the analyses of all stems and of juveniles alone, soils were also significant predictors and the greater distance of their centroids from the origin indicates that they have a greater effect on community composition than location on or off mounds.

**Table 2:** Results of canonical correspondence analysis of species distributions.

(a) Density of juveniles and adults		
Variable	Lambda	P
LA	0.54	0.007
DA	0.47	0.154
MOUND	0.47	0.257
WA	0.45	0.225

(b) Density of juveniles		
Variable	Lambda	P
LA	0.55	0.008
DA	0.49	0.087
MOUND	0.46	0.324
WA	0.46	0.197

(c) Density of adults		
Variable	Lambda	P
LA	0.99	0.062
MOUND	0.81	0.544
SH	0.77	0.699
WA	0.76	0.68

(d) Total basal are of juveniles and adults		
Variable	Lambda	P
MOUND	0.65	0.002
LA	0.6	0.015
DA	0.6	0.015
WA	0.53	0.047

(e) Total basal area of juveniles		
Variable	Lambda	P
MOUND	0.61	0.007
WA	0.59	0.001
LA	0.54	0.022
DA	0.49	0.118

(f) Total basal area of adults		
Variable	Lambda	P
LA	0.99	0.115
DA	0.79	0.435
MOUND	0.72	0.457
WA	0.59	0.855

Results based on the density of trees per m2 for each species with dbh >1 cm in the 2005 census still alive in 2008 are shown for (**a**) all stems (>1 cm dbh) (**b**) juveniles (1–10 cm dbh) (**c**) adults (>10 cm dbh). Results based on the total basal area per species per site are shown for (**d**) all stems (**e**) juveniles and (**f**) adults. The four soil types (wet alluvium (WA), dry alluvium (DA), shale (SH) and latterite (LA)) and location (on or off mounds) were used as nominal environmental variables. Surface areas of the ground and the mound were used as covariables. Lambda is the corresponding eigenvalue and P is the conditional probability level. With a Bonferroni correction for multiple comparisons, p is significant at the level of 0.008 (p = 0.05/6).

## Discussion

Our study investigated whether epigeal termite mounds affect tree abundance, diversity and community composition at a microhabitat scale in a lowland dipterocarp forest. We found that seedling species diversity was higher on mounds than in associated non-mound quadrats, but there was no difference in seedling density. We also found that mounds had significantly higher tree stem (>1 cm dbh) densities, but significantly lower species diversity than areas immediately surrounding mounds for all stems and considering juveniles alone. Moreover, we found that location on or off mounds significantly influenced community composition when species were quantified by basal area, both for all stems and when juveniles were considered separately. These results suggest that epigeal termite mounds may be providing a source of spatial heterogeneity that could contribute to the high tree species diversity in the Pasoh forest 50-ha plot.

We did not find any significant differences in mound distribution or structure between soil types, which indicates that overall soil properties do not govern the availability of epigeal mounds as a microhabitat for plants at Pasoh. These results are consistent with those of Abe and Matsumoto [43] who found a uniform dispersion of epigeal termite mounds in Pasoh Forest Reserve.

We did not find any significant difference in seedling density between mound and non-mound areas, but mound tree stem densities were significantly higher for all stems and for juveniles considered alone. This suggests mound soil properties result in higher levels of tree establishment, possibly through higher rates of seedling to sapling survival. McComie and Dhanrajan [24] report enriched nutrient levels in the internal chambers of *M. carbonarius* epigeal termite mounds in comparison with adjacent soils. The internal chambers are located in the above ground portion of a mound. It is therefore possible that the enriched nutrient levels of the mounds positively affect the establishment of young trees on these mounds.

Mounds had significantly higher diversity of seedling species than seedling plots located 3 m north of the mound. However, some caution is warranted as our seedling data were based on only eight mound – nonmound comparisons and the mound surfaces and non-mound quadrats were of unequal size. In contrast, mounds had a lower diversity of tree stems than the immediately surrounding non-mound areas, both for all stems and considering juveniles alone. The same trend was found when we compared the 3-m radius subplots with mounds and paired subplots without mounds. This is in contrast to results from drier ecosystems, such as a study on the effect of termite mounds on plant diversity in east African savanna, which found greater plant diversity in plots containing mounds than in plots without mounds [32]. Our results seem to suggest that mounds are suitable sites for initial establishment of a variety of species but that only a subset of species survive. It is possible that if the mounds contain higher levels of particular nutrients than the surrounding areas, then this might favor germination and establishment of large proportion of species. However, we might also expect that subsequent growth and survival would be dominated by a smaller number of species adapted to nutrient enriched conditions. This interpretation is also supported by the results of the canonical correspondence analysis.

Location on or off mounds was not a significant factor determining plant species distribution when the partial canonical correspondence analysis was based on densities of species. This indicates that there were no differences in species assemblages between mounds and the areas immediately surrounding mounds based on the number of stems. However, when the ordination was based on total basal area per species, location on or off mounds was a highly significant predictor of species distributions both when considering all stems and for juvenile stems alone. Enriched nutrient levels may result in faster growth on mounds for particular species and therefore detectable differences in species total basal area between mound and non-mound areas. The results taken together suggest that epigeal mounds do not affect the tree community that establishes on mounds up to the point of recruitment into the plot dataset (≥1 cm dbh), but do affect the subsequent survival and growth, which is also consistent with our results for stem densities and diversity reported above. Our finding that the relationship was significant for juvenile stems alone demonstrates that these results were not driven by the basal areas of a few large trees.

Our investigation focused on whether heterogeneity in soil nutrient or micro-topographic properties generated by mound building termites influenced the distribution and abundance of tree species at Pasoh. We investigated this at two spatial scales. At the finest scale we compared the superior portion of epigeal mounds with their immediate surroundings. The superior portion of epigeal mounds are easy to delimit in the field and present a distinct micro-topographic and nutrient enriched environment [24]. As described above, at this scale we found significant differences in seedling diversity, stem density (>1 cm dbh) and diversity and species composition on a basal area basis. Thus the superior portions of epigeal termite mounds do appear to provide a distinct micro-habitat that affects the distribution of tree species at Pasoh. We also compared 3-m radius circles with mounds and 3-m radius circles without mounds. The area around a mound will be affected to some degree through erosion of the mound surface and though termite tunneling activities in the subsurface portion of the mound. Hence, we might anticipate some affect of mounds at this scale. However, we were not able to detect any significant differences at this scale in the parameters we measured. It may be that given the abundance of termite mounds at Pasoh, our non-mound areas were not entirely “termite free”, either through past occupation or through the effects of erosion from nearby mounds. At the outset of our research, we were also interested in asking whether termite mounds might affect the abundance or diversity of tree stems at a 20×20 m plot scale. However, as a result of the high prevalence of epigeal mounds we were unable to find sufficient 20 m study plots without mounds. The relatively fine-grained distribution of termite mounds at Pasoh (31.25 mounds per ha) suggests that any effect on the abundance and diversity of tree species may only be evident at smaller spatial scales.

Detailed analyses of species-specific responses to epigeal mounds are the next step in understanding the role of epigeal mounds as a microhabitat for certain species in these forests. We attempted to test whether individual species exhibited differences in growth and mortality rates on and off mounds, but despite the large number of plots we surveyed our per species sample sizes were too small. We also investigated whether we could identify mound specialist species, but unfortunately again the per-species abundances in our dataset were too low. Ideally, one would track both seedling and mound dynamics over time. We suggest future studies look at the functional traits of species growing on and off of mounds in addition to species identity. We also recommend that future studies consider whether mounds are inhabited by termites as occupation has been shown to suppress plant growth in some systems [48]. The identity of the termite species may also be important in systems with more than one large-mound building termite species present. Finally, we suggest that future work focus on the physiological mechanisms involved through seedling experiments using mounds and mound soil.
